# Rising COVID-19 related acute pulmonary emboli but falling national chronic thromboembolic pulmonary hypertension referrals from a large national dataset

**DOI:** 10.1183/23120541.00431-2021

**Published:** 2021-10-11

**Authors:** Joseph Newman, Iryna Boubriak, David Jenkins, Choo Ng, Alessandro Ruggiero, Nicholas Screaton, John Cannon, Mark Toshner

**Affiliations:** 1 Royal Papworth Hospital, Cambridge, UK; 2 Dept of Medicine, Heart Lung Research Institute, University of Cambridge, Cambridge, UK

## Abstract

Chronic thromboembolic pulmonary hypertension (CTEPH) is an uncommon but significant complication of acute pulmonary embolism (PE). There is a strong, documented causal relationship between COVID-19 and venous thromboembolism. Despite this, we have observed a seemingly dichotomous reduction in the rate of new CTEPH referrals during the pandemic to our national specialist centre. Royal Papworth Hospital, Cambridge, is the UK's only quaternary CTEPH centre and captures all new diagnoses.

## Abstract 2

**The average rate of new #CTEPH referrals has dropped by 32% in the UK during the pandemic, despite the high incidence of #COVID19 related pulmonary emboli. There have been no recorded new cases of CTEPH caused by COVID-19. A prospective study is underway.** https://bit.ly/37msP2G

*To the Editor:*

Chronic thromboembolic pulmonary hypertension (CTEPH) is an uncommon but significant complication of acute pulmonary embolism (PE). There is a strong, documented causal relationship between COVID-19 and venous thromboembolism. Despite this, we have observed a seemingly dichotomous reduction in the rate of new CTEPH referrals during the pandemic to our national specialist centre. Royal Papworth Hospital, Cambridge, is the UK's only quaternary CTEPH centre and captures all new diagnoses.

We examined monthly referral rates of adult patients with confirmed CTEPH over the first 12 months of the COVID-19 pandemic (March 2020–February 2021). These data were compared to the 3 years prior (March 2017–February 2020). 1228 referrals were reviewed. Baseline referral rate was an average of 27.8 *versus* 19.0 per month during the pandemic phase (p<0.001) (figure 1). It can be expected from this that ∼106 cases of CTEPH in the UK and Ireland have not been diagnosed or referred during the pandemic.

**FIGURE 1:**
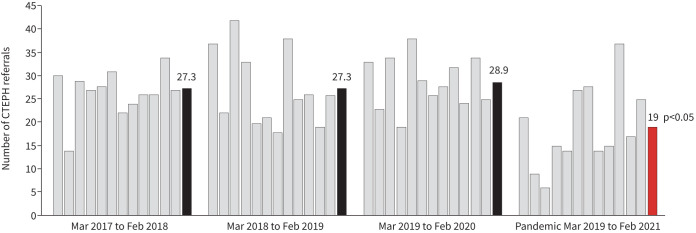
Monthly chronic thromboembolic pulmonary hypertension (CTEPH) referral rates with 12-month means highlighted for 3 years pre-pandemic compared to 12 months during the COVID-19 pandemic.

Comparing the demographics of the CTEPH cohorts, there was an even sex balance: 53.1% male during the pandemic, unchanged from baseline (51.4%, p=0.64). The mean age of the patients during the pandemic was unchanged: 60.6 years from a baseline of 60.9 years (p=0.83). During the pandemic, patients were more likely to be referred directly on from the CTEPH multidisciplinary team meeting for review and consideration of pulmonary endarterectomy (PEA), the definitive management of CTEPH (60.1% *versus* 49.6%, p<0.01). The reasons for this are likely to include selected referral of more urgent or severe cases, limited access to diagnostics in referring centres and an attempt to limit the number of hospital attendances.

There is clear evidence here that all seven pulmonary hypertension units in the UK and Ireland were still referring patients with CTEPH to the quaternary centre and in similar proportions to the baseline data. Clinicians should be reassured, therefore, that the pulmonary hypertension network is operating as “business as usual” during the pandemic, albeit with each centre receiving fewer referrals from regional hospitals.

By March 2021, there had been >4 million confirmed cases of COVID-19 in the UK, of which >446 795 patients have been hospitalised [1]. Using a very conservative estimate of incidence from the literature of 3.5% of hospitalised patients with COVID-19 developing acute PE [2], we would expect ∼15 638 excess annual PE diagnoses over the past year. In the pre-pandemic era, at least 1–3% of patients with PE would be expected to later develop CTEPH [3], giving a conservative estimate of 156 new cases of CTEPH.

It has not been established whether the acute PE seen with COVID-19 is mechanistically distinct and whether this extrapolation is justified. The lack of increase in CTEPH referrals may reflect a different pathophysiology. Indeed, none of the 228 CTEPH cases referred during the pandemic had a history or evidence of COVID-19. That said, the median time to CTEPH diagnosis from index PE has historically been 12.5 months [4], so the authors are continuing to track the referrals prospectively to see if COVID-19-related cases do emerge over the next 12 months.

Mechanistically, endothelial dysfunction may develop due to viral inclusion or the pro-inflammatory state and tissue hypoxia [5]. Simultaneously, pro-coagulant factors increase, platelets are activated and fibrinolysis reduces. CTEPH, by comparison, is thought to result primarily from incomplete breakdown of PE with organisation of the obstructing material into vessel walls and obstruction of blood flow through the pulmonary vasculature [6]. Given that this mechanism originates from an embolic event, arguably, it should not matter where or how the primary thrombosis occurs. There are two caveats to this. Firstly, there is evidence that CTEPH occurs in the absence of PE, as a primary *in situ* thrombotic phenomenon6; it is unclear how COVID-19 relates to this process. Secondly, the anatomy, size and distribution of acute clot burden influence CTEPH risk. CTEPH risk is thought to be greater with sub-massive emboli causing right ventricular strain, as has been studied in the large PEITHO prospective cohort [7]. COVID-19, in comparison, is more associated with microthrombotic distal lesions [5]. This would also influence management decisions, as PEA surgery is typically performed on large proximal lesions [8].

The decline in CTEPH referral rate is likely to be due to overwhelming pressure on the UK's National Health Service. Figure 1 shows that the lowest referral rate corresponds with the UK's “first wave” in April and May of 2020 before showing signs of recovery towards baseline. The recommended 3-month PE follow-up clinics [3] may have been postponed or cancelled. Access to diagnostics such as computed tomography imaging or nuclear medicine may be limited. Physicians working outside of their usual settings can lack the training or awareness of CTEPH to suspect this rare condition or may have assumed that referral services were suspended. Patients have been wary of attending hospital investigations during the pandemic. With these institutional, staffing and patient factors, it is likely that CTEPH has not have been carefully screened for. An international survey of patients with established (predominantly idiopathic) pulmonary arterial hypertension has reported on the disruption of their healthcare during the pandemic [9].

At 3-month follow-up, it is challenging to differentiate on-going symptoms, predominantly breathlessness, of thromboembolic disease from other COVID-19-related sequelae.

It is possible that the baseline incidence of non-COVID-19 related provoked PE has declined over the past year, although national data on this are lacking. At least two significant risk factors for the development of acute venous thromboembolism have been reduced. It is estimated that in the UK, 43 407 operations per week were cancelled [10] and global airline flight numbers were over 90% lower during the first wave [11]. This is likely to have had only a modest effect though and these risk factors for PE are distinct from those of CTEPH [8].

Overall, we suspect that the rate of CTEPH referrals has incongruously fallen due to the unprecedented pressure on the healthcare system, with hundreds of cases likely to have gone undiagnosed. It is possible that the pulmonary emboli seen with COVID-19 are mechanistically distinct or more amenable to anticoagulant treatment without the long-term complication of CTEPH. We strongly encourage clinicians to be mindful of this rare condition and refer suspected cases of CTEPH to specialist centres as per usual established guidelines and pathways, as untreated disease carries a poor prognosis, with a 5-year survival of only 53% [12].
